# Cardiac Myxoid Spindle Cell Tumor in a Neonate

**DOI:** 10.1155/2024/8630268

**Published:** 2024-06-26

**Authors:** Reza Abbaszadeh, Fatemeh Naderi, Amir Hossein Jalali, Yaser Toloueitabar

**Affiliations:** Rajaie Cardiovascular Medical and Research Center Iran University of Medical Sciences, Tehran, Iran

## Abstract

*Introduction*: Different subtypes of cardiac tumors containing spindle cells have been described as cardiac sarcoma. However, benign types have not been reported so far. We described a neonate with progressive respiratory distress who had a PDA and was finally diagnosed with a right atrial spindle cell tumor. *Case Presentation: *The patient was a neonate referred with respiratory distress and sepsis. The initial echocardiography demonstrated a small atrial septal defect, patent ductus arteriosus, and a heterogeneous rounded right atrial mass lesion. Pathologic examination confirmed the right atrial myxoid spindle cell tumor without local invasion. Successful mass resection was performed, and follow-up echocardiography revealed normal cardiac structure and function. *Conclusion*: In infants with manifestations of possible cardiac anomalies, it is necessary to consider other pathologies, such as neoplastic processes. Spindle cell detection in pathology is not ominous all the time, and there are benign subtypes with favorable outcomes after successful surgical resection.

## 1. Introduction

Myxoid soft-tissue tumors consist of a wide variety of mesenchymal lesions characterized by producing extracellular myxoid matrix and include a group of benign, aggressive, and even malignant tumors that may affect any soft-tissue organs [1]. The presence of considerable overlap in the clinical and pathological evidence of these lesions has made it difficult to reach a final diagnosis, especially when these lesions are traced in unusual areas. Spindle cell lesions are a wide spectrum of disorders ranging from reactive tumor-like masses to aggressive and poor malignant tumors [2]. Most of these tumors take source from mesenchymal tissues in nature; however, in some cases, there is a possibility of these lesions being neoplastic, and sometimes morphologically, instead of epithelioid morphological appearance, they have the morphology of spindle-shaped cells [3]. In general, the presence of myxoid masses with spindle-cell morphology is rare, and in particular, very few cases of this type of lesion have been reported in the soft tissues of the cardiovascular system. In the histopathologic evaluation, myxomas are seen as yellowish, white, or brownish masses with a pedunculated appearance. A thrombus can frequently cover these tumors, and their size ranges between one and ten centimeters in most cases [4]. Herein, we described a case of neonatal spindle cell tumor originating from the right atrium (RA).

## 2. Case Presentation

**Figure 1:**
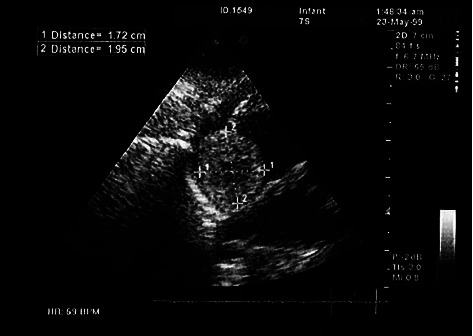
The echocardiography view of the lesion (a heterogeneous rounded atrial mass lesion (1.72 ✕ 1.95 cm^2^)).

The patient was a male neonate with a gestational age of 39 weeks born through natural vaginal delivery with an Apgar score of 9 to 10 at birth. The baby's birth weight was 3480 grams, but the neonate suffered from progressive respiratory distress that required intubation and surfactant administration. Within hospitalization, a blood culture was requested that was positive for coagulase-negative staphylococci. Initially, the patient was diagnosed with respiratory distress and sepsis and was scheduled for antibiotherapy (ampicillin, vancomycin), total parenteral nutrition, sildenafil, and ibuprofen. Echocardiography at birth revealed mild tricuspid regurgitation (TR), small atrial septal defect (ASD), and patent ductus arteriosus (PDA). In further echocardiography assessment, there was evidence of mild RA enlargement with a heterogeneous rounded mass lesion (1.72 ✕ 1.95 cm) within the RA cavity. Furthermore, a left ventricular (LV) ejection fraction of 50%, moderate TR, mild mitral regurgitation (MR), PDA (2.6 mm), and normal right ventricular (RV) function were detected (Figure 1). The appearance of the mass suggested the possibility of atrial myxoma. Therefore, the patient was a candidate for surgical resection. At surgery, the patient underwent sternotomy and total thymectomy. After heparin injection and reaching proper activating clotting time, the aorta, inferior vena cava (IVC), and superior vena cava (SVC) were cannulated and cardiopulmonary bypass (CPB) started. IVC and SVC were snared. The PDA was closed from inside the main pulmonary artery (MPA), and MPA was repaired. The aorta was clamped, and cardiac arrest was induced. The RA was opened. There was a firm mass in the lateral atrial wall extending to the IVC and anterior to it. A wide resection of the mass (2 ✕ 2 cm) was performed with a safe margin up to the edge of the suprahepatic veins. The resection site was repaired using an autologous pericardial patch. After repairing the atriotomy site and deairing, the aortic clamp was removed and the patient was disconnected from the CPB after rewarming. The rest of the operation was done as routine, and the patient was transferred to the ICU with stable vital signs.

**Figure 2:**
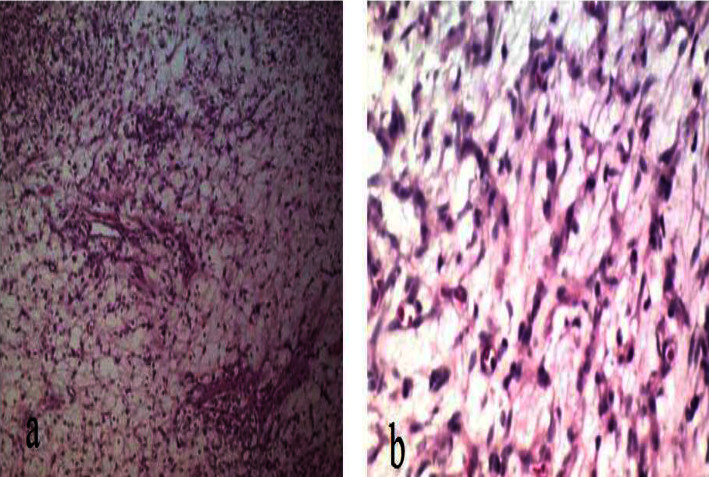
(a) Microscopic examination (H&E stain) shows that cellular neoplastic tissue contains bland-looking spindle cells in myxoid stroma with some small- and medium-sized vascular channels with scattered inflammatory cells. (b) Cellular atypia and necrosis are not seen.

**Figure 3:**
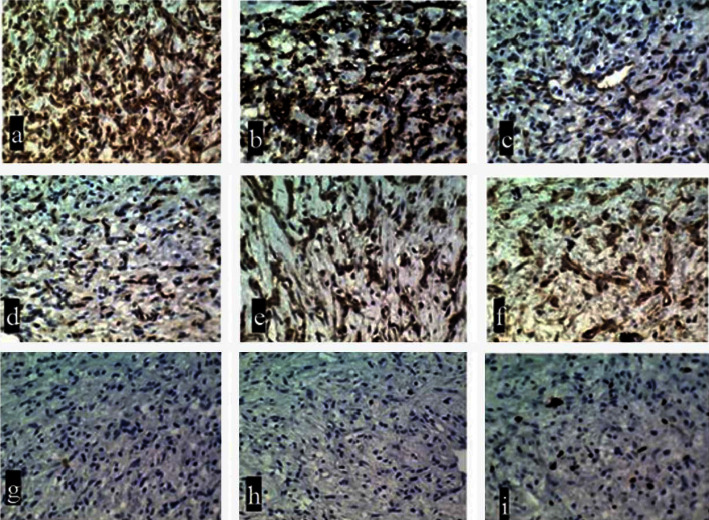
Immunohistochemical studies show positive reaction with vimentin (a), CD34 (b), CD31 (c), factor VIII (d), desmin (e), smooth muscle actin (f), ERG (not shown) and negative reaction with S100 (g), and calretinin (h). KI67 is positive in about 15% of tumor cells (i).

**Figure 4:**
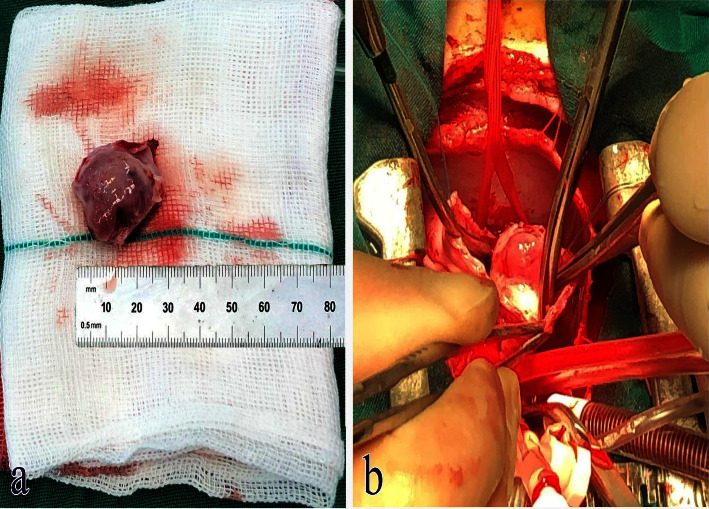
Macroscopic image of mass after surgical removal (a) and intraoperative view of the mass (b).

In pathological assessment, a cellular neoplastic tissue containing bland-looking spindle cells in the myxoid stroma was revealed. Medium and some large-sized vascular channels, congestion, and scattered inflammatory cells were also seen. No atypia or mitoses were noted (Figures 2 and 3). Intraoperative and postresection evaluation of the tumor confirmed the radiologic size and macroscopic features (Figure 4). In this regard, the definite diagnosis of RA myxoid spindle cell tumor was confirmed. The postoperative course was uneventful, and the patient was discharged with a good clinical condition.

Follow-up echocardiography after 1.5 years demonstrated no RA mass, mild TR, normal size RA, mild left atrial enlargement, no residual PDA, tricuspid annular plane systolic excursion of 12 mm, and normal LV function (LVEF of 55%). During the follow-up three years after the initial evaluations, ultrasound and CT scan of the abdomen and pelvis did not show any evidence of malignant involvement or extracardiac manifestations related to metastasis, so the lesion was confirmed to be benign.

## 3. Discussion

Different subtypes of benign and malignant tumors containing spindle cells have been described. These types of lesions are histologically characterized by a mixture of fibroblast-like spindle cells and sometimes fat cells in the field of myxoid, mucoid, or collagenous materials that can be tracked in different soft tissue organs such as the nervous system, gastrointestinal tract, breast, mesenchymal tissues, and even exocrine and endocrine glands [5]. Some rare patterns of these lesions have also been revealed in bony structures as well as in retroperitoneal space [6, 7]. Due to its rarity, a few studies have been performed on its clinical and pathological behaviors.

Spindle cell tumors were primarily described by Weiss et al. in 1896 as gastrointestinal stromal tumors, desmoid tumors, and fibrosarcoma [8]. Most of these tumor types were presented in younger ages and even in children, regardless of the patient's gender. Since the initial description of the biological behavior of tumors, their benign and malignant types (mostly sarcoma) have been described. The clinical manifestations of these tumors are very different depending on their location as well as their biological behavior but are generally nonspecific. Sometimes, they have been discovered only in radiological evaluations. However, in general, the final diagnosis of such tumors is possible based on histopathological evaluation and observing bland-looking spindle cells. In histological assessment, some specific staining, such as *β*-catenin nuclear staining, and immunohistochemical staining of some specific markers, such as CD34, CD117, and Ki-67, can be helpful in achieving a final diagnosis [8]. On the other hand, the malignant type of myxoma is rarely seen, and although they have enhanced mitotic activity and more prominent pleomorphism, differentiation of these types from the benign ones is not always straightforward with histologic findings. Malignancy features include causing peripheral aneurysms, regrowth in the primary location or other parts, and tumor invasion to adjacent structures [9–12]. None of these mentioned that hints of malignancy were seen in our case, implying the benign nature of the tumor.

In the management of such tumors, it is necessary to predict the behavior and biological nature of the mass in the first place and a different approach will be considered for benign and malignant masses. In the case of benign tumors, surgical resection is sufficient. However, extensive radical resection may be indicated in case of local invasion. Considering the risk of embolization and obstruction, cardiac myxoma treatment is surgical removal. Surgical removal is a simple, safe, and minimally invasive procedure that has demonstrated excellent short- and long-term outcomes and insignificant morbidity and mortality in several studies [13–16]. Moreover, although asymptomatic at the beginning, these tumors can cause severe morbidity and mortality in older children and adolescents [13, 17–19].

Most of the reported cases of cardiac spindle cell tumors have been related to sarcoma [20–22]. To our knowledge, this was the first case of myxoid type in cardiac tissue. The described case was noteworthy for several reasons. Our case was a neonate with progressive respiratory distress who had a PDA. Although cardiopulmonary anomalies or infections were the most probable diagnoses, a precise echocardiographic exam revealed chamber enlargement and an intraatrial mass. This observation emphasizes our awareness of rare conditions that could be present in association with more prevalent diagnoses. Moreover, we found that some tumors may have a similar gross appearance to cardiac myxoma, the most common primary cardiac tumor. Thus, an exact histopathologic evaluation is of great value in planning therapeutic strategies according to the tissue diagnosis. As we noticed, rare diagnoses other than rhabdomyoma or myxoma should not make us afraid. In our report, complete mass removal with no recurrence in the 1.5-year follow-up was excellent proof of the benign nature of the mass.

## Data Availability

All patients' data and documents can be available for the editorial if required.
